# Prediction of breast cancer risk based on flow variant analysis of circulating peripheral blood mononuclear cells

**DOI:** 10.1016/j.xhgg.2022.100085

**Published:** 2022-01-08

**Authors:** Johnny Loke, Ishraq Alim, Sarah Yam, Susan Klugman, Li C. Xia, Dorota Gruber, David Tegay, Andrea LaBella, Kenan Onel, Harry Ostrer

**Affiliations:** 1 Department of Pathology, Albert Einstein College of Medicine, Bronx, NY 10416, USA; 2 Morgan and Mendel Genomics, Bronx, NY 10461, USA; 3 Department of Obstetrics and Gynecology and Women’s Health, Montefiore Medical Center, Bronx, NY 10416, USA; 4 Department of Pediatrics, Cohen’s Children Medical Center, Northwell Health, New Hyde Park, NY 11040, USA; 5 Department of Human Genetics and Genomics, Icahn School of Medicine at Mount Sinai, New York, NY 10029, USA

**Keywords:** breast cancer, genetic testing, high risk, panel sequencing, functional genomics, variants of uncertain significance

## Abstract

Identifying women at high risk for developing breast cancer is potentially lifesaving. Patients with pathogenic genetic variants can embark on a program of surveillance for early detection, chemoprevention, and/or prophylactic surgery. Newly diagnosed cancer patients can also use the results of gene panel sequencing to make decisions about surgery; therefore, rapid turnaround time for results is critical. Cancer Risk B (CR-B), a test that uses flow variant assays to assess the effects of variants in the DNA double-strand break repair, was applied to two groups of subjects who underwent coincidental gene panel testing, thereby allowing an assessment of sensitivity, specificity and accuracy, and utility for annotating variants of uncertain significance (VUS). The test was compared in matched peripheral blood mononuclear cells (PBMCs) and lymphoblastoid cells (LCLs) and tested for rescue in LCLs with gene transfer. The CR-B phenotype demonstrated a bimodal distribution: CR-B^+^ indicative of DSB repair defects, and CR-B^−^, indicative of wild-type repair**.** When comparing matched LCLs and PBMCs and inter-day tests, CR-B yielded highly reproducible results. The CR-B^−^ phenotype was rescued by gene transfer using wild-type cDNA expression plasmids. The CR-B^−^ phenotype predicted VUS as benign or likely benign. CR-B could represent a rapid alternative to panel sequencing for women with cancer and identifying women at high risk for cancer and is a useful adjunct for annotating VUS.

## Introduction

For women at high risk for developing breast cancer (MIM: 114480), gene panel sequencing identifies cancer-predisposing pathogenic or likely pathogenic (P/LP) variants in 15%–20% and variants of uncertain significance (VUS) in ≥40%.^1^, ^2^, ^3^, ^4^ The remainder of those tested have no detectable genetic alteration; these patients as well as those with VUS have no reduction of risk. Thus, >80% of women are left with uncertainty regarding their risk for developing breast cancer. To fill the gap, we developed flow variant assays (FVAs) that assess the nuclear localization and phosphorylation of proteins in the DNA double-strand break (DSB) repair pathway following challenge with radiomimetic agents that trigger the pathway.^5^^,^^6^ These assays identified defects in BRCA1 and BRCA2 nuclear localization and p53 phosphorylation in lymphoblastoid and circulating B cells from individuals with P/LP variants in *BRCA1* (MIM: 113705)*, BRCA2* (MIM: 600185), and other genes in the DSB repair pathway. The FVAs showed that most VUS in these genes did not disrupt the pathway and are benign or likely benign (B/LB). A risk classification score based on logistic regression of these three FVAs performed on circulating B cells was >90% accurate for defects in the pathway. The resulting test, Cancer Risk B (CR-B), identified defects in the pathway even when no causal variants could be found by whole-genome sequencing.

Here, we present a simplified version of the test with comparable sensitivity and specificity that can be performed on whole-blood peripheral mononuclear cells (PBMCs) without prior enrichment for B cells. Comparable results were obtained for PBMCs and lymphoblastoid cells (LCLs) derived from the same individual. The CR-B^+^, high-risk phenotype associated with P/LP gene-specific variants in LCLs was rescued by expression of the wild-type (WT) gene following plasmid transfection, demonstrating the causality of these variants.

## Material and methods

### Subjects

Subjects were recruited from cancer genetic counseling programs at Montefiore Medical Center (Table S1) and Northwell Health (Table S2) under approved institutional review board protocols. Montefiore recruitment took place from May 8, 2018 through April 9, 2019, while Northwell recruitment occurred from October 21, 2019 through December 26, 2020. All of the subjects were women older than 18 years of age, had undergone gene panel sequencing for breast cancer risk based on National Comprehensive Cancer Network (NCCN) criteria,^7^ and provided informed consent for deidentified CR-B testing and chart review. Single variant testing was offered to relatives of subjects found to have P/LP variants in DSB repair genes (*BRCA1*, *BRCA2*, *PALB2* [MIM: 610355], *BARD1* [MIM: 601593], *RAD51C* [MIM: 602774], *RAD51D* [MIM:602954], *CHEK2* [MIM: 604373], and *ATM* [MIM: 607585]) per NCCN criteria.^7^ Those found to be negative for the P/LP variant were included in estimates of specificity. Among the information shared were age; personal cancer diagnosis and treatment; family history of breast, ovarian (MIM: 167000), or other cancers; and results of gene panel tests. PBMCs from individuals were transformed to LCLs to compare the performance of FVAs for these two cell types over the range of positive (N = 10) and negative (N = 10) risk classification scores (RCSs). LCLs with P/LP variants or VUS and negative RCSs were selected to test the effects of expression plasmid rescue. LCLs from the National Institute of General Medical Sciences genetic disease database and the 1000 Genomes Project, previously studied by CR-B,^5^ were included as controls for FVAs and plasmid rescue.

### CR-B testing

CR-B testing was performed, as described previously.^5^ To analyze PBMCs, whole blood was cultured in the presence of radiomimetic agents. Cells were lysed partially to obtain a mix of nuclei and intact cells for analysis, then stained with DAPI, and antibodies conjugated with fluorochromes fluorescein isothiocyanate (FITC), PECy7, APCCy7, and PECy5.5. Flow cytometry was performed using a BD Canto II (BD Biosciences, Franklin Lakes, NJ) equipped with blue (488 nm), red (638 nm), and violet lasers (407 nm) in a 4-2-2 configuration.

The results were analyzed using FlowJo software (FlowJo, Ashland, OR). The RCS was calculated using logistic regression coefficients for each of the assays, as described previously.^5^ The RCS was normalized, so that the log of the odds ratio of pathogenic to benign was zero at the equal likelihood. The coefficient of variation (CV) was calculated for three replicates of each individual sample. Correlation coefficients were calculated for the same samples run on different days or for PBMCs and LCLs derived from the same subjects. Sensitivity was calculated as CR-B^+^ subjects with P/LP variants in DSB repair genes divided by all subjects with P/LP variants, and specificity was calculated as CR-B^−^ relatives not inheriting P/LP variants divided by all relatives not inheriting P/LP variants. Boxplots and Mann-Whitney tests were performed to identify potential differences in FVAs between subjects from different groups (positive controls with known P/LP variants, negative controls from related family members without P/LP variants, subjects with and without breast or ovarian cancer diagnoses). Recommendations for the use of functional assays to annotate genetic variants were applied to the CR-B^−^ phenotype as a Bayesian conditional probability model,^8^^,^^9^ as well as an independent model to determine how it would affect the reclassification of specific VUS.

### Gene rescue

Expression plasmids (1 μg) for BRCA1 (pDEST-FRT/T0-GFP-BRCA1, cat. no. 71116, GFP tag), BRCA2 (pMH-SFB-BRCA2, cat. no. 99395, SFP tag), PALB2 (pDEST-FRT/T0-GFP-PALB2, cat. no. 71113, GFP tag), and ATM (pcDNA3.1(+)FLAG-His-ATM WT, cat. no. 31985, Addgene, Watertown, MA) were transfected into WT LCLs or those with P/LP or B/LB variants. The constructs without cDNA inserts (1 μg) were used for sham transfection to demonstrate specificity of the rescue. All of the transfections were performed by electroporation with a LifeTech Neon Transfection system used following the recommendations of the manufacturer (Thermo Fisher Scientific, Waltham, MA; MPK 5000). The efficiency of transfection could be gauged by the number of cells expressing the in-frame reporter (GFP, SBP, or FLAG-His). Following transfection, the cells were treated according to the standard CR-B protocol and then assessed with CR-B. For those expression plasmids that interfere with FITC (i.e., GFP), AM-CYAN was used with S6 instead of the FITC standard in CR-B. Boxplots and Student’s t tests were performed to identify the potential differences in rescue.

## Results

### The CR-B test performs comparably in PBMCs and LCLs and has high sensitivity and specificity

**Figure 1:**
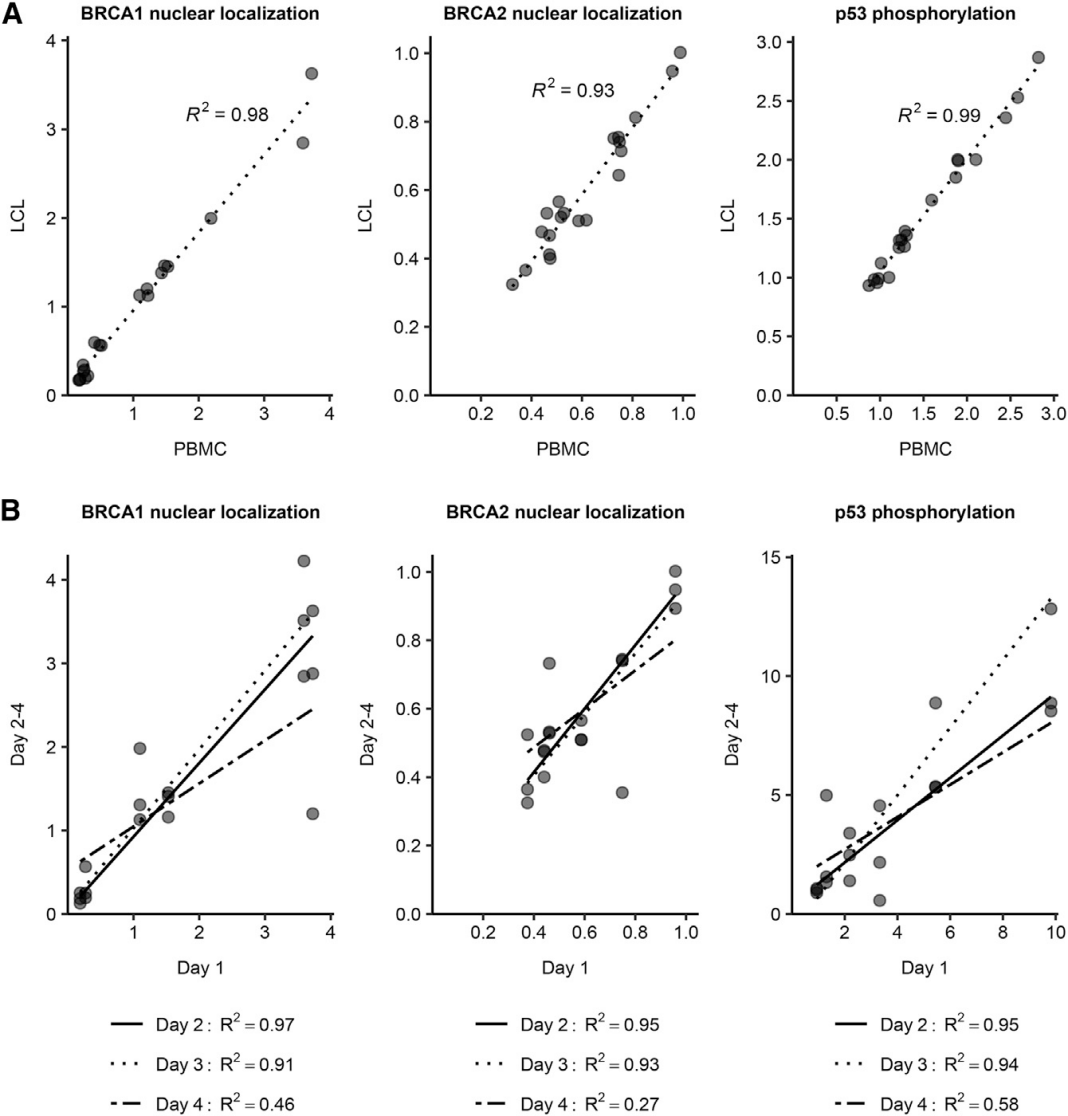
Replication of CR-B FVAs (A) Replication of BRCA1 nuclear localization, BRCA2 nuclear localization, and p53 in matched LCLs and PBMCs derived from the same individuals (N = 20). Correlation cofficients are shown. (B) Replication of BRCA1 nuclear localization, BRCA2 nuclear localization, and p53 phosphorylation of days 2–4 following sample collection, compared to day 1. Correlation cofficients are shown.

Previously, we showed that the CR-B test performed comparably in purified B cells and LCLs.^5^ To eliminate the need for cell purification, we compared FVAs performed on PBMCs in radiomimetic-treated and partially lysed whole blood to LCLs treated in the same way and derived from the same individuals. The results were highly reproducible for the individual FVAs (BRCA1 nuclear localization, r^2^ = 0.98; BRCA2 nuclear localization, r^2^ = 0.93; and p53 phosphorylation, r^2^ = 0.99; Figure 1A; Table S3). For the individual FVAs performed on LCLs and PBMCs, the mean CVs for triplicate analyses were <3% for BRCA1 nuclear localization, BRCA2 nuclear localization, and p53 ratio (Table S3), and only 3% of all PBMC replicates exceeded CV 3%. The individual FVAs performed on PBMCs were reproducible whether performed on days 1, 2, or 3 following collection, but not on day 4 (r^2^ > 0.9 for BRCA1 nuclear localization and BRCA2 nuclear localization, and p53 phosphorylation on days 2 or 3 compared to day 1; Figure 1B). Based on these observations, we transitioned the assays to whole-blood samples.

Previously, we reported that the sensitivity and specificity for the individual BRCA1 and BRCA2 nuclear localization and phospho-p53:total p53 ratio assays ranged from 82% to 93%, 91% to 93%, and 86% to 91%, respectively, and that the accuracy, based on the weighted mean of sensitivity and specificity for individual assays, ranged from 86% to 92%.^5^ When the RCS was calculated by combining these 3 assays using logistic regression, the sensitivity, specificity, and accuracy increased to 91%, 100%, and 94%, respectively. Applying the same approach to the Montefiore cohort, the sensitivity, specificity, and accuracy, based on known pathogenic variant heterozygotes in the *BRCA1* and *BRCA2* genes and known relatives testing negative for those variants, were 75%–100% for individual assays and 100% for the RCS (Table 1). No known relatives testing negative for pathogenic variants were recruited into the Northwell cohort, so specificity could not be calculated; however, the sensitivity was 75% for individual assays and the RCS. Combining results across studies, including those previously reported, the sensitivity, specificity, and accuracy of the individual assays ranged from 84% to 94%. For RCS, the sensitivity, specificity, and accuracy were 91%, 100%, and 95%, respectively.

**Table 1:** Sensitivity, specificity, and accuracy of FVAs and RCS for Coriell, Montefiore, and Northwell cohorts and all cohorts

Cohort	BRCA1	BRCA2	p53	RCS
Coriell (N = 36)^a^				
Sensitivity	0.82 (18/22)	0.91 (20/22)	0.91 (20/22)	0.91 (20/22)
Specificity	0.93 (13/14)	0.93 (13/14)	0.86 (12/14)	1 (14/14)
Accuracy	0.86 (31/36)	0.92 (33/36)	0.89 (32/36)	0.94 (34/36)
Montefiore (N = 6)				
Sensitivity	1 (2/2)	1 (2/2)	1 (2/2)	1 (2/2)
Specificity	1 (4/4)	0.75 (3/4)	0.75 (3/4)	1 (4/4)
Accuracy	1 (6/6)	0.83 (5/6)	0.83 (5/6)	1 (6/6)
Northwell (N = 4)				
Sensitivity	0.75 (3/4)	0.75 (3/4)	0.75 (3/4)	0.75 (3/4)
Specificity	NA	NA	NA	NA
Accuracy	NA	NA	NA	NA
All cohorts (N = 46)^b^				
Sensitivity	0.84 (38.5/46)	0.91 (41.7/46)	0.91 (41.7/46)	0.91 (41.7/46)
Specificity	0.94 (39.4/42)	0.90 (37.9/42)	0.84 (35.4/42)	1.00 (42/42)
Accuracy	0.88 (37/42)	0.90 (38/42)	0.88 (37/42)	0.95 (40/42)

a Coriell data were derived from Table S2.^5^

b All of the cohorts represent weighted results from individual cohorts.

### The CR-B phenotype demonstrates a bimodal distribution in high-risk individuals

**Figure 2:**
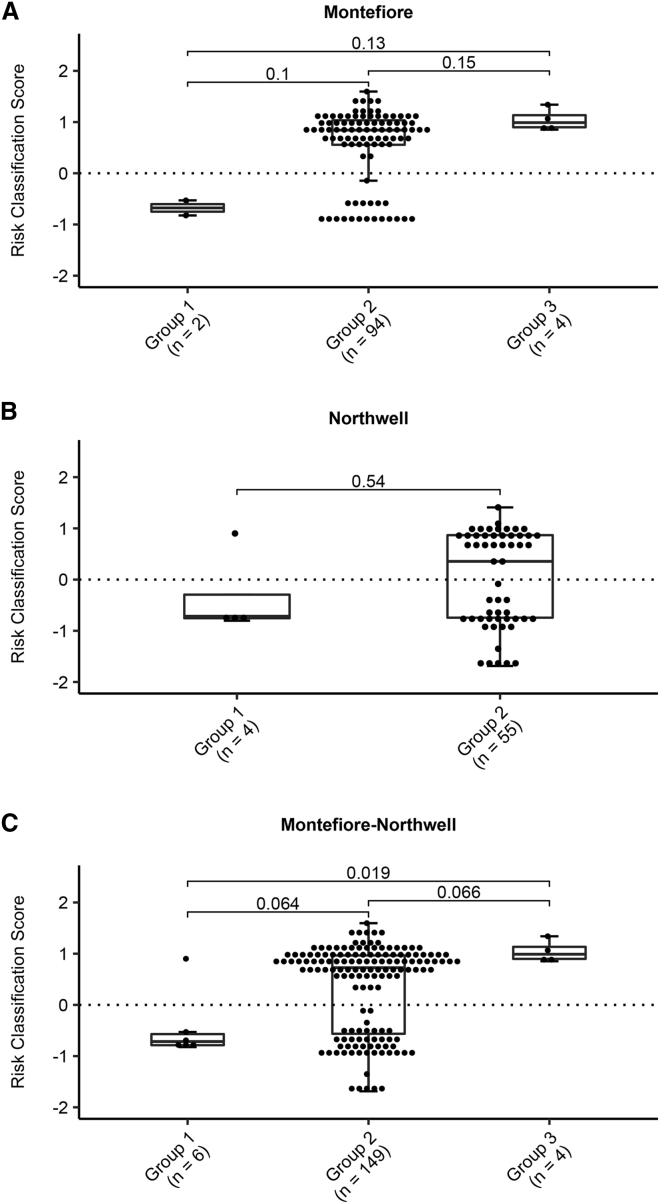
Bimodal distribution of CR-B RCS (A–C) Distribution of RCS in P/LP variant heterozygotes in DSB repair genes (group 1), VUS or no reported variants (group 2), and relatives testing negative for the familial variant (group 3) in (A) Montefiore cohort (N = 100), (B) Northwell cohort (N = 59), and (C) combined cohorts by boxplots (N = 159). P-values for pairwise comparisons are shown.

In a prior CR-B study, two distinct clusters, one CR-B^+^ and the other CR-B^−^, were described in high-risk subjects based on the individual FVAs and the resulting RCS.^5^ A similar phenomenon was observed among the Northwell and Montefiore cohorts (Figure 2; Tables S1 and S2). In the Northwell cohort, 30 subjects (50.8%) had RCS > 0 (CR-B^−^) and 29 subjects (49.2%) had RCS < 0 (CR-B^+^). Within the Montefiore cohort, 22 subjects (22%) had RCS > 0 (CR-B^−^) and 78 subjects (78%) had RCS < 0 (CR-B^+^). These findings suggest that CR-B is bimodal rather than continuously distributed, with CR-B^+^ subjects being at high risk for developing breast cancer and CR-B^−^ subjects reverting to population risk.

### The CR-B^−^ phenotype identifies VUS as B/LB

The VUS status in these two cohorts was confirmed in ClinVar and additional evidence for annotation was added (Table S4). The CR-B^−^ phenotype met all of the recommendations for collection and use of multiplexed functional data for clinical variant interpretation (Table S5)^10^ and for the application of the functional evidence PS3/BS3 categories using the American College of Medical Genetics and Genomics/Association for Molecular Pathology (ACMG/AMP) sequence variant interpretation framework (Table S6).^11^ Based on these criteria, the evidence for CR-B^−^ as a BS3 categorical classifier (high sensitivity, specificity, and reproducibility) is strong. The evidence for and against pathogenicity was entered into the recent Bayesian-adapted guidelines using the default values.^8^ In every instance, the variant was reannotated as B/LB (Table S4). When using these guidelines, assuming that the prior probability of any new variant being either benign or pathogenic was equal (prior = 0.50), every VUS was reannotated as LB. Applying the sensitivity (0.91) and specificity (0.96) over a range of prior probabilities indicated that all variants with prior ≤ 0.50 were reannotated as B/LB (Figure S1).

### The CR-B^+^ phenotype can be rescued by gene transfer in LCLs

**Figure 3:**
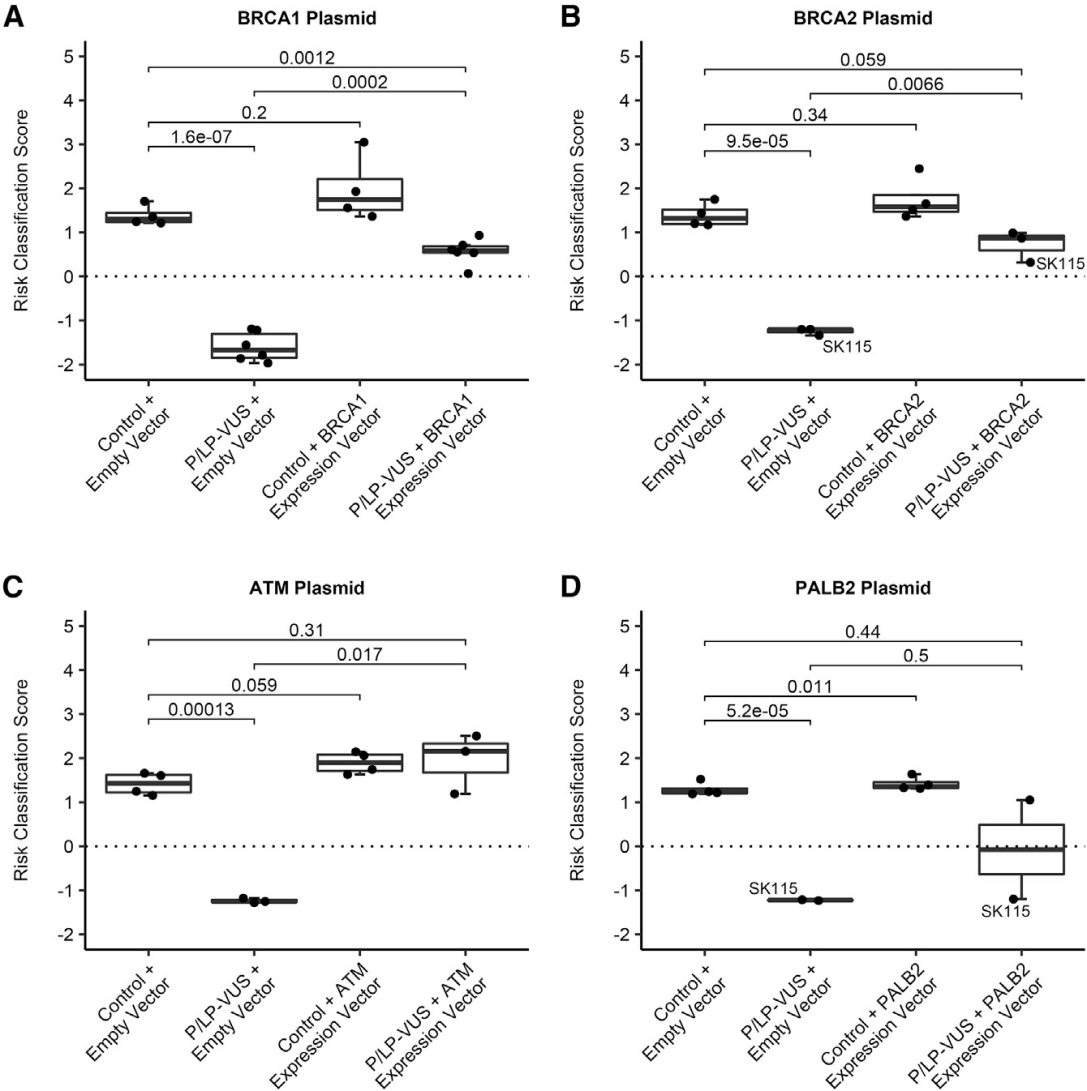
Expression plasmid rescue of genetic variants in LCLs (A–D) Gene rescue for (A) *BRCA1*, (B) *BRCA2*, (C) *ATM*, and (D) *PALB2* variants by RCS by boxplots. P-values for pairwise comparisons are shown.

Prior CR-B studies correlated the presence of P/LP variants in genes in the DSB repair pathway with the CR-B^+^ phenotype.^6^^,^^12^ To demonstrate the causality of these variants, we transfected LCLs with expression plasmids containing WT BRCA1, BRCA2, ATM, and PALB2 cDNAs or containing just the expression vector (Figures 3, S2, and S3; Table S7). Each vector had a reporter cDNA in frame to demonstrate expression in transfected cells. The overall efficiency of transfection exceeded 90%. These expression plasmids showed negligible effects when transfected into WT LCLs or LCLs with B/LP variants. When transfected into LCLs with P/LP variants, BRCA1 expression rescued *BRCA1* variants (p = 0.0002), BRCA2 expression rescued *BRCA2* variants (p = 0.0066), ATM expression rescued *ATM* variants (p = 0.017), and PALB2 expression rescued a *PALB2* variant (Figure 3). One LCL with VUS in both *BRCA2* and *PALB2* was rescued only by the BRCA2 cDNA and not by the PALB2 cDNA, thus demonstrating the *BRCA2* variant was causal for the CR-B^+^ phenotype. None of these expression plasmids rescued LCLs with P/LP variants in *NBN* (MIM: 602667) and *FANCI* (MIM: 611360) that were shown previously to be correlated with the CR-B^+^ phenotype (data not shown). These studies demonstrated the causality of specific P/LP variants for the CR-B^+^ phenotype and recapitulated the experiments of an earlier era when gene rescue was used to identify the genes that accounted for the different BRCA-FA complementation groups in homozygous/compound heterozygous cells.^13^

## Discussion

In this study, we performed the CR-B test on PBMCs derived from whole blood and demonstrated analytical validity of these assays by their reproducibility on successive days and their correlation with LCLs derived from the same subjects. Their clinical validity was demonstrated by the high sensitivity and specificity and by the gene rescue of LCLs with the CR-B^+^ phenotype using gene transfer for the known mutated gene.

The CR-B test fills a gap for those who fulfill NCCN guidelines for gene panel sequencing. Only 15%–20% of those tested are found to have P/LP variants (8% in Montefiore and 20% in Northwell). Of the remainder, 45% were found to have VUS (Montefiore: 18% DSB repair VUS, 34% all VUS; Northwell: 22% DSB repair VUS, 39% all VUS).^1^, ^2^, ^3^, ^4^ The currently followed practice of waiting for VUS to be reannotated by the testing laboratory could be supplanted by CR-B testing of these individuals. Virtually all VUS-bearing PBMCs that are CR-B^−^ could be reannotated as B/LB, because the test fulfills all of the recommendations for the use of multiplexed functional data for clinical variant interpretation.^10^ Notably, the analysis should be applied on an individual variant basis that uses all of the evidence for that variant to reclassify using strong functional evidence (BS3) in a Bayesian model.^8^^,^^14^ This approach would not be applicable to CR-B^+^ subjects because demonstrating causality by a VUS would require gene rescue of the phenotype by expression plasmid transfection, as demonstrated here.

This study does not offer guidance about odds ratios nor absolute risks for developing breast cancer as population-based and family-based case-control studies of panel sequencing have provided.^15^^,^^16^ Similar study designs could define the clinical utility of the test as a standalone by estimating breast cancer risks for CR-B^+^ individuals. Major advantages would be that virtually all individuals would be classified as CR-B^+^ or CR-B^−^, with few falling into the indeterminate zone, rapid completion and reporting of the test (within 2 days), and lower costs than those associated with gene panel library construction, sequencing, and DNA variant interpretation. Should the reported high sensitivity of CR-B be conserved over larger studies, it would meet the criteria favored by recent survey respondents to a new germline cancer risk assessment test: high sensitivity and specificity, rapid turnaround time, and low cost.^17^

CR-B represents a highly plausible test for identifying functionally important alterations in the DSB repair pathway. As shown in this study, CR-B is more sensitive and specific for identifying DSB repair defects, even when a variant cannot be identified by sequencing. The specificity of the test was demonstrated in gene rescue experiments. The identification of the CR-B^−^ population-risk phenotype in individuals with VUS reclassified these variants as B/LB using Bayesian analysis. The use of CR-B following gene panel sequencing resolved VUS and those that do not have variants to population risk for subjects found to be CR-B^−^ (BS3). Currently, the CR-B test could be used as an adjunct to gene panel sequencing.

## Data Availability

The data from this study are available in the supplemental tables.

## Web resources

CADD v1.6, https://cadd.gs.washington.edu/snv

ClinVar, https://www.ncbi.nlm.nih.gov/clinvar/

dbSNP, https://www.ncbi.nlm.nih.gov/snp/

OMIM, https://www.omim.org/

PolyPhen-2, http://genetics.bwh.harvard.edu/pph2/

PubMed, https://pubmed.ncbi.nlm.nih.gov/

SIFT, https://sift.bii.a-star.edu.sg/

## Supplemental information

Document S1. Figures S1–S3 and Tables S3–S7

Table S1. Montefiore cohort, including age, gender, genetic test result, CR-B FVAs, and RCS

Table S2. Northwell cohort, including age, gender, genetic test result, CR-B FVAs, and RCS

Document S2. Article plus supplemental information

## References

[1] Kurian A.W., Hughes E., Handorf E.A., Gutin A., Allen B., Hartman A.R., Ha M.J. (2017). Breast and ovarian cancer penetrance estimates derived from germline multiple-gene sequencing results in women. JCO Precis. Oncol..

[2] Couch F.J., Shimelis H., Hu C., Hart S.N., Polley E.C., Na J., Hallberg E., Moore R., Thomas A., Lilyquist J. (2017). Associations between cancer predisposition testing panel genes and breast cancer. JAMA Oncol..

[3] Hiraki S., Rinella E.S., Schnabel F., Oratz R., Ostrer H. (2014). Cancer risk assessment using genetic panel testing: considerations for clinical application. J. Genet. Couns..

[4] Easton D.F., Pharoah P.D., Antoniou A.C., Tischkowitz M., Tavtigian S.V., Nathanson K.L., Devilee P., Meindl A., Couch F.J., Southey M. (2015). Gene-panel sequencing and the prediction of breast-cancer risk. N. Engl. J. Med..

[5] Syeda M.M., Upadhyay K., Loke J., Pearlman A., Klugman S., Shao Y., Ostrer H. (2017). Prediction of breast cancer risk based on flow-variant analysis of circulating peripheral blood B cells. Genet. Med..

[6] Loke J., Pearlman A., Upadhyay K., Tesfa L., Shao Y., Ostrer H. (2015). Functional variant analyses (FVAs) predict pathogenicity in the BRCA1 DNA double-strand break repair pathway. Hum. Mol. Genet..

[7] Daly M.B., Pilarski R., Yurgelun M.B., Berry M.P., Buys S.S., Dickson P., Domchek S.M., Elkhanany A., Friedman S., Garber J.E. (2020). NCCN guidelines insights: genetic/familial high-risk assessment: breast, ovarian, and pancreatic, version 1.2020. J. Natl. Compr. Cancer Netw..

[8] Tavtigian S.V., Greenblatt M.S., Harrison S.M., Nussbaum R.L., Prabhu S.A., Boucher K.M., Biesecker L.G., ClinGen sequence variant interpretation working G. (2018). modeling the ACMG/AMP variant classification guidelines as a Bayesian classification framework. Genet. Med..

[9] Richards S., Aziz N., Bale S., Bick D., Das S., Gastier-Foster J., Grody W.W., Hegde M., Lyon E., Spector E. (2015). Standards and guidelines for the interpretation of sequence variants: a joint consensus recommendation of the American College of Medical genetics and genomics and the association for Molecular Pathology. Genet. Med..

[10] Gelman H., Dines J.N., Berg J., Berger A.H., Brnich S., Hisama F.M., James R.G., Rubin A.F., Shendure J., Shirts B. (2019). Recommendations for the collection and use of multiplexed functional data for clinical variant interpretation. Genome Med..

[11] Brnich S.E., Abou Tayoun A.N., Couch F.J., Cutting G.R., Greenblatt M.S., Heinen C.D., Kanavy D.M., Luo X., McNulty S.M., Starita L.M. (2019). Recommendations for application of the functional evidence PS3/BS3 criterion using the ACMG/AMP sequence variant interpretation framework. Genome Med..

[12] Loke J., Pearlman A., Radi O., Zuffardi O., Giussani U., Pallotta R., Camerino G., Ostrer H. (2014). Mutations in MAP3K1 tilt the balance from SOX9/FGF9 to WNT/beta-catenin signaling. Hum. Mol. Genet..

[13] Strathdee C.A., Gavish H., Shannon W.R., Buchwald M. (1992). Cloning of cDNAs for Fanconi’s anaemia by functional complementation. Nature.

[14] Brnich S.E., Rivera-Munoz E.A., Berg J.S. (2018). Quantifying the potential of functional evidence to reclassify variants of uncertain significance in the categorical and Bayesian interpretation frameworks. Hum. Mutat..

[15] Hu C., Hart S.N., Gnanaolivu R., Huang H., Lee K.Y., Na J., Gao C., Lilyquist J., Yadav S., Boddicker N.J. (2021). A population-based study of genes previously implicated in breast cancer. N. Engl. J. Med..

[16] Breast Cancer Association C., Dorling L., Carvalho S., Allen J., Gonzalez-Neira A., Luccarini C., Wahlstrom C., Pooley K.A., Parsons M.T., Fortuno C. (2021). Breast cancer risk genes - Association analysis in more than 113,000 women. N. Engl. J. Med..

[17] Klugman S., Schnabel F., Alim I., Loke J., Arun B., Chun Kim J., Ostrer H. (2021). Health care professionals' attitudes toward cancer gene panel testing. Breast J..

