# Supplementary material for: Prediction of breast cancer risk based on flow variant analysis of circulating peripheral blood mononuclear cells
Source: HGG Adv. 2022 Jan 8;3(2):100085. doi: 10.1016/j.xhgg.2022.100085 (PMC8801379; doi:10.1016/j.xhgg.2022.100085)
Supplement: Document S1. Figures S1–S3 and Tables S3–S7 [file mmc1.pdf]

HGGA, Volume *3*

## Supplemental information

# Prediction of breast cancer risk based on flow variant analysis of circulating peripheral blood mononuclear cells

**Johnny Loke, Ishraq Alim, Sarah Yam, Susan Klugman, Li C. Xia, Dorota Gruber, David Tegay, Andrea LaBella, Kenan Onel, and Harry Ostrer**

## Supplemental figures and tables

**Figure S1.** Application of Bayesian analysis for sensitivity 0.91 and specificity 0.99 over a range of prior probabilities.

**Figure S2.** Boxplots of "sham" or expression vector gene rescue in LCLs for A. *BRCA1* and B. *BRCA2* variants by individual CR-B FVAs. Pairwise comparisons were performed by Student t-tests.

**Figure S3.** Boxplots of "sham" or expression vector gene rescue in LCLs for A. *ATM* and B. *PALB2* variants by individual CR-B FVAs. Pairwise comparisons were performed by Student t-tests.

**Table S1.** Montefiore cohort, including age, gender, genetic test result, CR-B FVAs and RCS.

**Table S2.** Northwell cohort, including age, gender, genetic test result, CR-B FVAs and RCS.

**Table S3.** CR-B FVAs and coefficients of variation for matched LCL and PBMC samples from the same individuals in the Montefiore cohort.

**Table S4.** Reclassification of VUS in the Montefiore and Northwell cohorts based on CR-B- phenotype.

**Table S5.** Fulfillment of recommendations for the collection and use of multiplexed functional data for clinical variant interpretation.

**Table S6.** Recommendations for application of the functional evidence PS3/BS3 criterion using the ACMG/AMP sequence variant interpretation framework.

**Table S7.** Gene rescue for *BRCA1*, *BRCA2*, *ATM* and *PALB2* variants by individual FVAs.

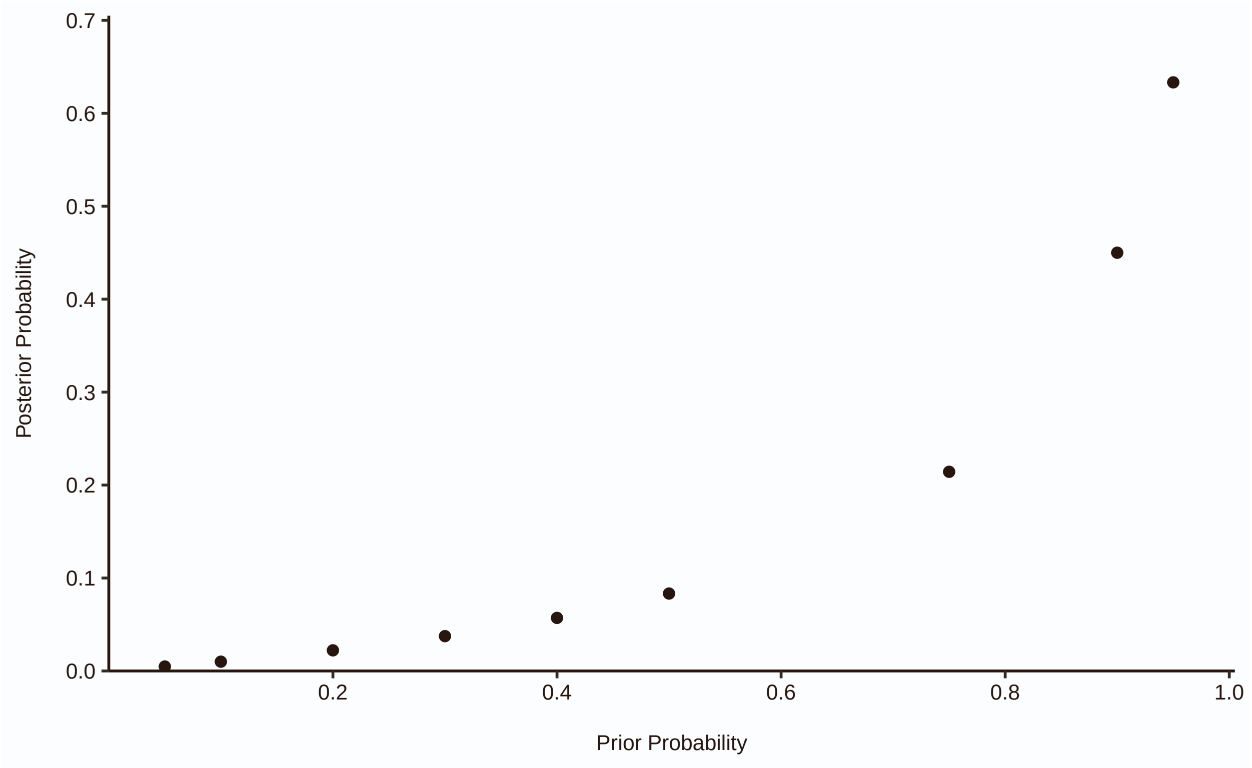


**Figure S1. Application of Bayesian analysis for sensitivity 0.91 and specificity 0.99 over a range of prior probabilities.**

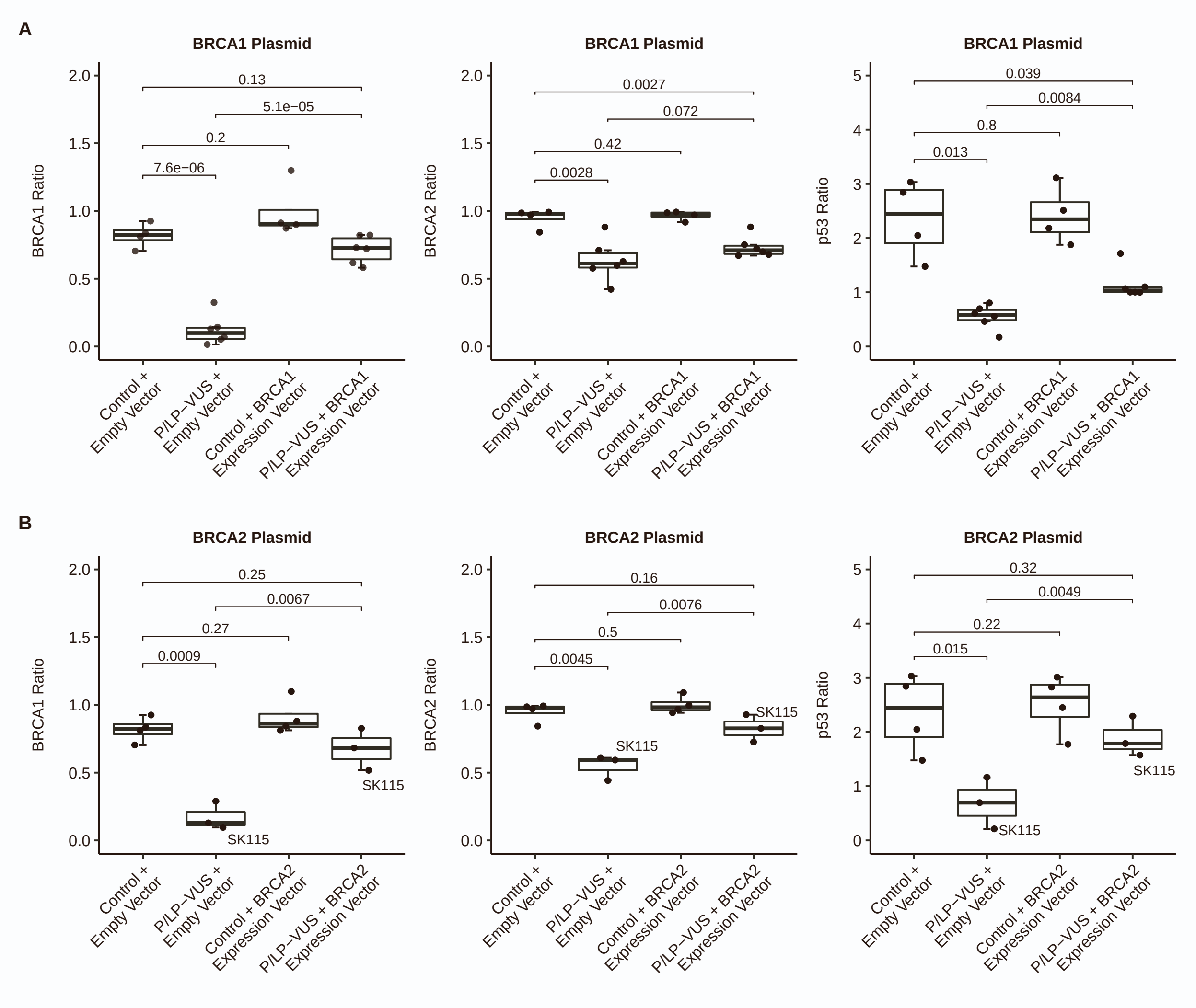


**Figure S2. Boxplots of "sham" or expression vector gene rescue in LCLs for A. *BRCA1* and B. *BRCA2* variants by individual CR-B FVAs.** Pairwise comparisons were performed by Student t-tests.

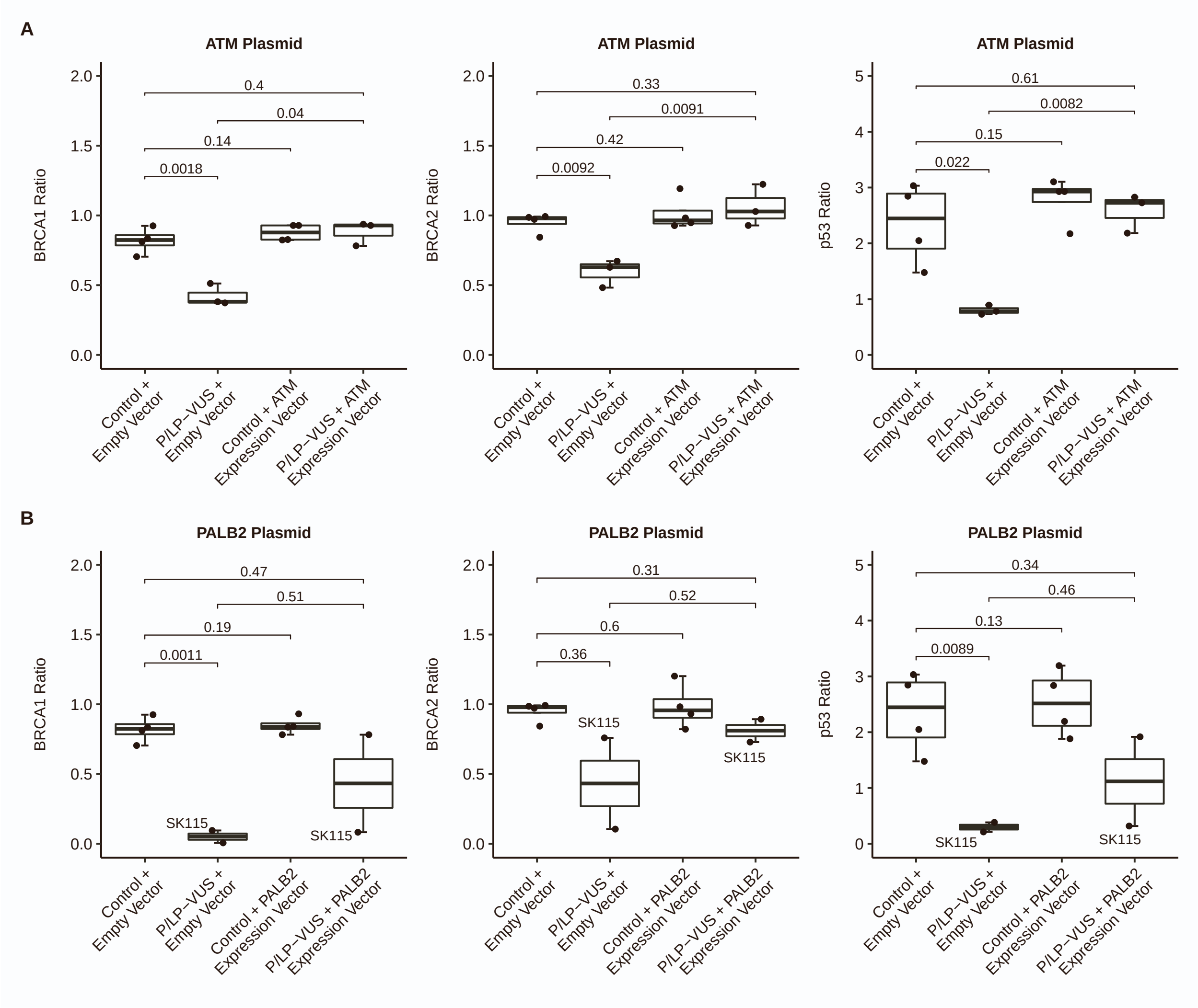


**Figure S3. Boxplots of "sham" or expression vector gene rescue in LCLs for A. *ATM* and B. *PALB2* variants by individual CR-B FVAs.** Pairwise comparisons were performed by Student t-tests.

**Table S3. CR-B FVAs and coefficients of variation for matched LCL and PBMC samples from the same individuals in the Montefiore cohort.**

| ID | Age | Gender | Genetic Panel Results | | PBMC | | | LCL | | | LCL Classification Score |
|---|---|---|---|---|---|---|---|---|---|---|---|
| | | | VUS | P/LP | BRCA1 Ratio | BRCA2 Ratio | p53 Ratio | BRCA1 Ratio | BRCA2 Ratio | p53 Ratio | |
| SK59 | 53 | F | | | 0.19 | 0.44 | 1.29 | 0.18 | 0.48 | 1.39 | -1.26 |
| SK44 | 33 | F | | | 3.59 | 0.75 | 2.82 | 2.85 | 0.74 | 2.87 | 1.07 |
| SK104 | 42 | F | | | 3.73 | 0.96 | 1.59 | 3.63 | 0.95 | 1.66 | -0.64 |
| SK70 | 34 | F | | | 1.53 | 0.75 | 1.30 | 1.45 | 0.64 | 1.36 | 0.94 |
| SKF1 | 42 | F | | NM_007294.4(BRCA1):c.68_69del | 1.10 | 0.46 | 2.45 | 1.13 | 0.53 | 2.36 | 0.37 |
| SK170 | 77 | F | | | 0.27 | 0.59 | 1.22 | 0.19 | 0.51 | 1.25 | -1.20 |
| SK46 | 46 | F | | NM_007294.4(BRCA1):c.5277+1del | 0.25 | 0.47 | 1.25 | 0.29 | 0.40 | 1.32 | -1.23 |
| SK123 | 37 | F | | NM_058216.3(RAD51C):c.211A>T | 1.23 | 0.74 | 2.58 | 1.12 | 0.75 | 2.53 | 0.74 |
| SK159 | 54 | F | | | 2.19 | 0.99 | 1.89 | 2.00 | 1.00 | 2.00 | 2.41 |
| SKP1 | 29 | F | | NM_000038.6(APC):c.3183_3187del | 0.41 | 0.32 | 0.93 | 0.60 | 0.32 | 0.98 | -0.91 |
| SKP3 | 48 | F | | NM_000051.3(ATM): c.(8936+1_8937-1)_(9137+1_9138-1) | 0.31 | 0.53 | 0.87 | 0.22 | 0.53 | 0.93 | -1.16 |
| SK69 | 40 | M | NM_000059.4(BRCA2):c.3860_3863del | | 0.48 | 0.51 | 0.97 | 0.57 | 0.57 | 0.96 | -0.58 |
| SK155 | 59 | F | | NM_007294.4(BRCA1):c.68_69del | 0.24 | 0.52 | 1.01 | 0.27 | 0.52 | 1.12 | -1.09 |
| SKP2 | 30 | F | | NM_000251.2(MSH2): c.(1795+1_1795-1)_(3115)del | 0.18 | 0.47 | 1.22 | 0.17 | 0.47 | 1.32 | -1.30 |
| SK171 | 58 | F | | NM_007294.4(BRCA1):c.68_69del | 0.17 | 0.62 | 1.28 | 0.17 | 0.51 | 1.27 | -1.23 |
| SK67 | 61 | F | NM_007294.4(BRCA1):c.3995G>T | | 0.51 | 0.47 | 0.98 | 0.56 | 0.41 | 0.99 | 1.09 |
| SK129 | 42 | F | | | 0.23 | 0.38 | 1.10 | 0.34 | 0.37 | 1.00 | 0.78 |
| SK64 | 34 | F | | | 1.21 | 0.73 | 1.87 | 1.20 | 0.75 | 1.85 | 0.78 |
| SK66 | 53 | F | | | 1.43 | 0.76 | 2.10 | 1.38 | 0.71 | 2.00 | 0.85 |
| SK135 | 44 | F | | | 1.48 | 0.81 | 1.90 | 1.46 | 0.81 | 1.99 | 1.29 |
| | | | **Mean FVA CV** | | 2.23 | 1.83 | 1.84 | 2.21 | 1.07 | 1.16 | |

**Table S4. Reclassification of VUS in the Montefiore and Northwell cohorts based on CR-B- phenotype.**

| VUS | Protein Change | dbSNP ID | CADD v1.6 | SIFT | PolyPhen-2 | Computational Majority[a] | CR-B | Submission Evidence[b] | Compiled Evidence[c] | Combined Odds_Path[d] | Post_P[d] | Interpretation[d] |
|---|---|---|---|---|---|---|---|---|---|---|---|---|
| ATM c.6176C>T | T2059I | rs144761622 | 18.07 | 0.04 | Inconsistent across GRCh38/37 | - | CR-B- (4), CR-B+ (1) | | BS3, PP3 | 0.11 | 0.012 | LB |
| ATM c.7855A>G | S2619G | - | 19.49 | 0.78 | 0 | Benign | CR-B- | | BS3, BP4 | 0.03 | 0.003 | B |
| ATM p.Gln368Ile | Q368I | - | - | - | - | - | CR-B- | | BS3 | 0.05 | 0.006 | B |
| BARD1 c.1913C>T | A638V | rs1559374246 | 23.4 | 0.06 | 0.857 | Deleterious | CR-B- | | BS3, PP3 | 0.11 | 0.012 | LB |
| BARD1 c.977A>G | N326S | rs779960429 | 0.001 | 1 | 0.003 | Benign | CR-B- | PMID: 30925164 [BS3] | BS3, BP4 | 0.03 | 0.003 | B |
| BRCA1 c.3995G>T | G1332V | rs730881490 | 18.43 | 0.04 | 0.105 | Deleterious | CR-B- | PMID: 15343273, 26320175 [PM1] | BS3, PP3 | 0.11 | 0.012 | LB |
| BRCA2 c.5342A>G | D1781G | rs80358756 | 13.71 | 0.14 | 0.645 | Benign | CR-B- | PMID: 12491487 [BP4] | BS3, BP4 | 0.03 | 0.003 | B |
| BRIP1 c.2220G>T | Q740H | rs45589637 | 22.1 | 0.02 | 1 | Deleterious | CR-B- | PMID: 25186627, 25980754 [BP5] | BS3, PP3, BP5 | 0.05 | 0.006 | B |
| BRIP1 c.2255A>G | K752R | rs876660016 | 21.9 | 0.33 | 0.149 | Benign | CR-B- | | BS3, BP4 | 0.03 | 0.003 | B |
| BRIP1 c.2284C>T | R762C | rs587778136 | 32 | 0 | 0.987 | Deleterious | CR-B- | | BS3, PP3 | 0.11 | 0.012 | LB |
| BRIP1 c.3571A>G | I1191V | rs761405340 | 7.067 | 0.63 | 0 | Benign | CR-B- | | BS3, PP3 | 0.11 | 0.012 | LB |
| CHEK2 c.59A>G | Q20R | rs753257724 | 23.2 | 0.01 | 0.932 | Deleterious | CR-B- | | BS3, PP3 | 0.11 | 0.012 | LB |
| PALB2 c.3404G>A | G1135E | rs730881894 | 26.1 | 0 | 1 | Deleterious | CR-B- | LabCorp America internal data [BP5], PMID: 31636395 [BS3] | BS3, PP3, BP5 | 0.05 | 0.006 | B |
| RAD50 c.2177G>A | R726H | rs28903092 | 24.7 | 0.03 | 0.346 | Deleterious | CR-B- | Located in Zinc Hook domain, Uniprot: IPR013134 [PM1] | BS3, PP3, PM1 | 0.11 | 0.012 | LB |
| RAD50 c.259C>T | R87C | rs143802516 | 23.8 | 0.01 | 0.024 | Deleterious | CR-B- | Located in the SbcC-type AAA, PMID: 18466635 [PM1] | BS3, PP3, PM1 | 0.11 | 0.012 | LB |
| RAD50 c.3640C>T | R1214C | rs546479838 | 32 | 0 | 1 | Deleterious | CR-B- | Invitae internal data [BP2] | BS3, BP2, PP3 | 0.05 | 0.006 | B |
| RAD51C c.134A>G | E45G | rs587781383 | 24.1 | 0.15 | 0.003 | Benign | CR-B- | Located in region required for Holliday junction resolution activity involved in homologous recombination, Uniprot: O043502 [PM1] | BS3, BP4, PM1 | 0.11 | 0.012 | LB |

Scores are interpreted by computational method as pathogenic (orange)

Scores are interpreted by computational method as benign (green)

[a]Computational majority calculated as majority interpretation across CADD v1.6, SIFT, and PolyPhen-2 scores.
[b]PubMed, ClinVar, and Uniprot citations for variant
[c]ACMG scores[9]
[d]Combined odds pathogenic, posterior probability, and interpretations[8]

**Table S5. Fulfillment of recommendations for the collection and use of multiplexed functional data for clinical variant interpretation.**

| Recommendation[10] | Met |
|---|---|
| 1. Assays must have sufficient dynamic range to separate robustly disease-relevant, functionally abnormal variant classes such as loss- or gain-of-function from functionally normal variants. | Yes |
| 2. Choose an assay design and model system that can assess the type of variant associated with disease. | Yes |
| 3. Report data sets using FAIR standards, including the reporting of raw data such as sequencing reads and variant counts. | Yes |
| 4. To standardize terminology, report variant scores using the ENIGMA variant-effect ontology. | Yes |
| 5. Include each target's stable, versioned accession number from a common genomic database. | Yes |
| 6. Conduct replicates to characterize the reliability of assay results. | Yes |
| 7. Variants from across the full range of assay scores should be tested singly in the same and/or orthogonal functional assay such that a quantitative measure of consistency can be calculated and reported. | Yes |
| 8. List all pathogenic and benign variants chosen for [assay] validation along with their database of origin and, if possible, their accession numbers or publication references. | Yes |
| 9. Report the predictive value of multiplexed functional data in terms of sensitivity and specificity. | Yes |
| 10. The strength of evidence that can be provided by the multiplexed functional data should be determined on a variant-by-variant basis that accounts for both the error associated with the measurements for the specific variant and the overall trustworthiness and predictive power of the assay. | Yes |
| 11. Do not stack evidence from multiple [assays] for the same variant | N/A |

**Table S6. Recommendations for application of the functional evidence PS3/BS3 criterion using the ACMG/AMP sequence variant interpretation framework.**

| | Recommendation[11] | Met |
|---|---|---|
| 1. | Define the disease mechanism | Yes |
| 2. | Does the general class of assay model pathogenesis/disease mechanism? | Yes |
| 3. | Evaluate validity of specific instances of assay | Yes |
| 4. | Apply evidence to individual variant interpretation | Yes |

**Table S7. Gene rescue for *BRCA1*, *BRCA2*, *ATM* and *PALB2* variants by individual FVAs.**

| Variant | ID | Clinical Significance[a] | Sample Source[b] | Un-rescued | | | Rescued | | |
|---|---|---|---|---|---|---|---|---|---|
| | | | | BRCA1 Ratio | BRCA2 Ratio | p53 Ratio | BRCA1 Ratio | BRCA2 Ratio | p53 Ratio |
| NM_007294.4(BRCA1):c.4065_4068del | SK138 | P | Montefiore | 0.0151 | 0.5772 | 0.6111 | 0.6172 | 0.6712 | 1.716 |
| NM_007294.4(BRCA1):c.6187_6197del | SKP4 | P | Montefiore | 0.1293 | 0.7101 | 0.6962 | 0.7298 | 0.7512 | 1.0661 |
| NM_007294.4(BRCA1):c.3995G>T, NM_177438.2(DICER1):c.1867C>T | SK67 | VUS, VUS | Montefiore | 0.3246 | 0.8810 | 0.4625 | 0.8212 | 0.8817 | 1.0027 |
| NM_007294.4(BRCA1):c.68_69del | GM14090 | P | NIGMS | 0.1420 | 0.4220 | 0.8036 | 0.5819 | 0.7221 | 1.0027 |
| NM_007294.4(BRCA1):c.5266dup | GM14091 | P | NIGMS | 0.0528 | 0.5981 | 0.5577 | 0.7221 | 0.6988 | 1.0001 |
| NM_007294.4(BRCA1):c.181T>G | GM14097 | P | NIGMS | 0.0701 | 0.6274 | 0.1707 | 0.8221 | 0.6788 | 1.1003 |
| Control | GM19084 | B | HapMap | 0.7043 | 0.9860 | 2.8440 | 0.9123 | 0.9872 | 2.1829 |
| Control | HG01812 | B | 1000 Genomes | 0.8119 | 0.9718 | 3.0330 | 0.8728 | 0.9928 | 3.1128 |
| Control | HG02621 | B | 1000 Genomes | 0.8355 | 0.8434 | 2.0483 | 1.2991 | 0.9173 | 2.5122 |
| Control | HG02884 | B | 1000 Genomes | 0.9255 | 0.9922 | 1.4767 | 0.8999 | 0.9712 | 1.8773 |
| NM_000059.3(BRCA2):c.125A>G | GM14623 | B* | NIGMS | 0.1293 | 0.6101 | 0.6962 | 0.6827 | 0.9281 | 1.7882 |
| NM_000059.3(BRCA2):c.5342A>G | SK132 | VUS | Montefiore | 0.2890 | 0.4418 | 1.1630 | 0.8271 | 0.7261 | 2.2918 |
| NM_000059.3(BRCA2):c.7712A>G, NM_024675.3(PALB2):c.1337A>T | SK115 | VUS, VUS | Montefiore | 0.0955 | 0.5931 | 0.2126 | 0.5173 | 0.8272 | 1.5732 |
| Control | GM19084 | B | HapMap | 0.7043 | 0.9860 | 2.8440 | 0.8123 | 0.9421 | 2.8290 |
| Control | HG01812 | B | 1000 Genomes | 0.8119 | 0.9718 | 3.0330 | 0.8428 | 0.9681 | 3.0128 |
| Control | HG02621 | B | 1000 Genomes | 0.8355 | 0.8434 | 2.0483 | 1.0991 | 1.0917 | 2.4512 |
| Control | HG02884 | B | 1000 Genomes | 0.9255 | 0.9922 | 1.4767 | 0.8799 | 0.9971 | 1.7727 |
| NM_000051.3(ATM):c.6404_6405insTT | GM01525 | P | NIGMS | 0.5122 | 0.4823 | 0.7291 | 0.7821 | 0.9281 | 2.1827 |
| NM_000051.3(ATM):c.7913G>A | GM03332 | P | NIGMS | 0.3812 | 0.6281 | 0.8927 | 0.9372 | 1.0281 | 2.8271 |
| NM_000051.3(ATM):c.7913G>A | GM03334 | P | NIGMS | 0.3729 | 0.6721 | 0.7821 | 0.9281 | 1.2230 | 2.7261 |
| Control | GM19084 | B | HapMap | 0.7043 | 0.9859 | 2.8439 | 0.8239 | 0.9271 | 3.1029 |
| Control | HG01812 | B | 1000 Genomes | 0.8119 | 0.9717 | 3.0329 | 0.8271 | 1.1920 | 2.9281 |
| Control | HG02621 | B | 1000 Genomes | 0.8355 | 0.8433 | 2.0483 | 0.9281 | 0.9821 | 2.9281 |
| Control | HG02884 | B | 1000 Genomes | 0.9255 | 0.9922 | 1.4766 | 0.9281 | 0.9471 | 2.1721 |
| NM_000059.3(BRCA2):c.7712A>G, NM_024675.3(PALB2):c.1337A>T | SK115 | VUS, VUS | Montefiore | 0.0955 | 0.7593 | 0.2126 | 0.0828 | 0.7291 | 0.3182 |
| NM_024675.3(PALB2):c.844_847del | SK16 | P | Montefiore | 0.0066 | 0.1051 | 0.3835 | 0.7821 | 0.8931 | 1.9172 |
| Control | GM19084 | B | HapMap | 0.7043 | 0.9859 | 2.8439 | 0.78212 | 1.2019 | 2.8371 |
| Control | HG01812 | B | 1000 Genomes | 0.8119 | 0.9717 | 3.0329 | 0.8361 | 0.9821 | 3.1928 |
| Control | HG02621 | B | 1000 Genomes | 0.8355 | 0.8433 | 2.0483 | 0.8412 | 0.8212 | 2.1928 |
| Control | HG02884 | B | 1000 Genomes | 0.9255 | 0.9922 | 1.4766 | 0.9312 | 0.9312 | 1.8821 |

[a]Pathogenic (P), Variant Unknown Significance (VUS), Benign (B)

[b]HapMap and 1000 Genomes Project samples are housed by the National Human Genome Research Institute (NHGRI) Sample Repository for Human Genetic Research. National Institute of General Medical Sciences (NIGMS) Human Cell Repository and NHGRI samples were collected through the Coriell Institute for Medical Research.

*Individual had CR-B+ results, early onset of breast cancer and family history of cancer. Panel sequencing only showed a BRCA2 variant, classified as Benign by ClinVar. Of tested WT plasmids (BRCA1, BRCA2, PALB2, and ATM), only BRCA2 showed rescue.
